# Preparation of Protein–Peptide–Calcium Phosphate Composites for Controlled Protein Release

**DOI:** 10.3390/molecules25102312

**Published:** 2020-05-14

**Authors:** Katsuya Kato, Sungho Lee, Fukue Nagata

**Affiliations:** National Institute of Advanced Industrial Science and Technology (AIST), 2266-98 Anagahora, Shimoshidami, Moriyama-ku, Nagoya 463-8560, Japan; sungho.lee@aist.go.jp (S.L.); f.nagata@aist.go.jp (F.N.)

**Keywords:** calcium phosphate, protein, peptide, controlled release, hydroxyapatite

## Abstract

Protein–peptide–calcium phosphate composites were developed for achieving sustainable and controlled protein release. Bovine serum albumin (BSA) as a model acidic protein was efficiently encapsulated with basic polypeptides such as polylysine and polyarginine during the precipitation of calcium phosphate (CaP). The prepared composites were fully characterized in terms of their morphologies, crystallinities, and the porosity of their structures, and from these analyses, it was observed that there are no significant differences between the composites. Scanning transmission electron microscopy and energy dispersive X-ray spectroscopy analysis indicated a homogeneous distribution of nitrogen and sulfur, confirming the uniform distribution of BSA and polypeptide in the CaP composite. In vitro release studies demonstrated that the composite prepared with the peptides α-polylysine and polyarginine were suitable for the gradual release of the protein BSA, while those containing ε-polylysine and no peptide were unsuitable for protein release. Additionally, these composites showed high hemocompatibility for mouse red blood cells, and the osteoblast-like cell proliferation and spread in media with the composites prepared using BSA and α-polylysine showed similar tendencies to medium with no composite. From these results, protein–peptide–CaP composites are expected to be useful as highly biocompatible protein delivery agents.

## 1. Introduction

A drug delivery system (DDS) is a method used to administer (bio)pharmaceutical compounds such as nucleic acids and proteins into the human body to achieve a therapeutic effect, and can improve the efficacy or reduce the toxicity of anti-inflammatory and anti-cancer agents [1,2,3,4,5]. A wide number of materials have been used in drug delivery, including polymers and organic–inorganic composites such as bioactive glasses or metal oxides [6,7]. Recently, a stimuli-responsive DDS that responds to certain external stimuli, such as temperature and pH, has been used as a promising way of increasing the effectiveness of chemotherapeutics [8,9]. For example, it is well known that the extracellular pH of normal cells (pH = 7.0–7.5) is more basic than that of cancer cells (pH = 4.5–6.5); moreover, numerous pH gradients exist in the intracellular compartment where cells uptake a DDS carrier material during endocytosis. Therefore, there is the need to develop DDS carriers that can retain their drug cargo under neutral conditions for long periods before sustainably releasing the said drug under slightly acidic conditions. To date, there has been growing interest in organic (polymer and liposomes) and inorganic materials (metal oxides and metal phosphates) as carriers to release the active drug by environmental changes such as pH and temperature, which represents a promising approach for the treatment of a range of different diseases, although these systems are still under investigation [10,11].

Calcium phosphate (CaP), a major inorganic component of human bone, is widely used in a range of clinical applications due to its extremely high biocompatibility [12,13]. CaP materials and their polymer composites are useful as bone and tooth substitutes, and due to their good bioactivity are used as tissue scaffolds. Additionally, CaP nanomaterials are used as gene transfection carriers using nucleic acids and as materials for theranostics using enzymes [14,15,16,17,18,19]. CaP materials exhibit higher biocompatibility and biodegradability than other inorganic materials such as metal oxides (silica and titania), carbon nanotubes, and quantum dots, and dissolve under slightly acidic pH (pH = 5) conditions to form biocompatible ionic constituents (Ca^2+^ and PO_4_^3−^). This aspect means that they have great potential for application as carriers of various drugs, including biomolecules [20,21].

CaP–organic composites are very attractive for use in many applications, not only as tissue-engineering scaffolds and bone graft substitutes, but they are also useful in bioseparation, biosensing, and biocatalysis [22,23]. Inzana et al. [24] reported that maximum flexural strength and cell viability was increased on 3D printed scaffolds designed using CaP–collagen composites. Hadisi et al. [25] found that CaP–starch–silk fibroin nanofibers prepared using an electrospinning method improved the viability, proliferation, and attachment of osteoblast-like cells. Nonoyama et al. [26] investigated self-assembled β-sheet peptides formed using a CaP–peptide hydrogel and found that these could be used as injectable bone-filling materials. Previously, we reported CaP formation on β-sheet peptide-monolayer templates with surface carboxyl groups and found that the CaP morphology and crystallinity were dependent on the peptide sequences [27]. We also designed α-helix and β-sheet peptide templates containing L-glutamic acid for CaP mineralization [28], where the prepared CaP–peptide composites exhibited protein adsorption with high selectivity for basic proteins (e.g., cytochrome c and lysozyme). Very recently, we observed [29] that glucose oxidase immobilized on poly(L-lysine)-containing CaP particles exhibited high activity and that their relative activity remained at approximately 70% after ten cycles. In addition, poly (L-glutamic acid)-containing CaP showed high adsorption for the basic protein avidin and its effective binding to biotin, with a theoretical value of four (biotin/avidin molecular ratio) [30]. From these results, it was determined that peptide–CaP composites have great potential as carriers of biomolecules for use in DDS.

In this study, the in situ encapsulation of protein–peptide hybrids were investigated during the precipitation of CaP. The resulting protein–peptide–CaP composites were fully characterized in terms of their particle morphologies, crystallinities, and surface and porous structures. Their protein release profiles exhibited that the release rate could be controlled according to the structures of the peptides. The biocompatibility of the protein–peptide–CaP composites was also investigated in detail. To the best of our knowledge, the formation and characterization of protein–peptide–CaP composites and their controllable delivery of protein have not yet been reported in the literature.

## 2. Results and Discussion

### 2.1. Preparation of the Protein–Peptide–CaP Composites and Their Characterization

In our previous report [31], we described the synthesis of peptide–calcium phosphate composites and their ability to adsorb proteins. These composites exhibited highly selective adsorption characteristics for various proteins, and the amount of protein loading on the composites was found to vary depending on the structures of the peptides. In addition, the enzymes adsorbed on the composites exhibited higher or comparable catalytic activities to those of free enzymes in solution [29]. As an extension of our previous work, the in situ encapsulation of proteins during the preparation of a peptide–CaP composite was developed for various biological applications. 

First, fluorescein isothiocyanate–bovine serum albumin conjugate (FITC–BSA, isoelectric point; pI: approximately 4.7) was added to a solution mixture of basic polypeptide (poly-α-lysine, αpLys; poly-ε-lysine, εpLys; or poly-α-arginine, pArg) and calcium ions to enable the peptides and calcium ions to adsorb on the surface of the FITC–BSA molecules. After adding the phosphate ions, CaP immediately formed and a protein–peptide material was simultaneously encapsulated inside the CaP structure. As a preliminary experiment, FITC–BSA was encapsulated in the peptide–CaP composite. Figure S1 shows the fluorescence spectra of the protein solution before CaP precipitation and the supernatant after CaP precipitation. The fluorescence spectrum of FITC–BSA was recorded using a spectrofluorophotometer at an excitation wavelength of 280 nm and fluorescence wavelengths of 300–440 nm. No fluorescence intensity was observed from the solution after collecting the precipitate, indicating that all of the BSA molecules were effectivity encapsulated during the formation of the peptide–CaP composite.

**Figure 1:**
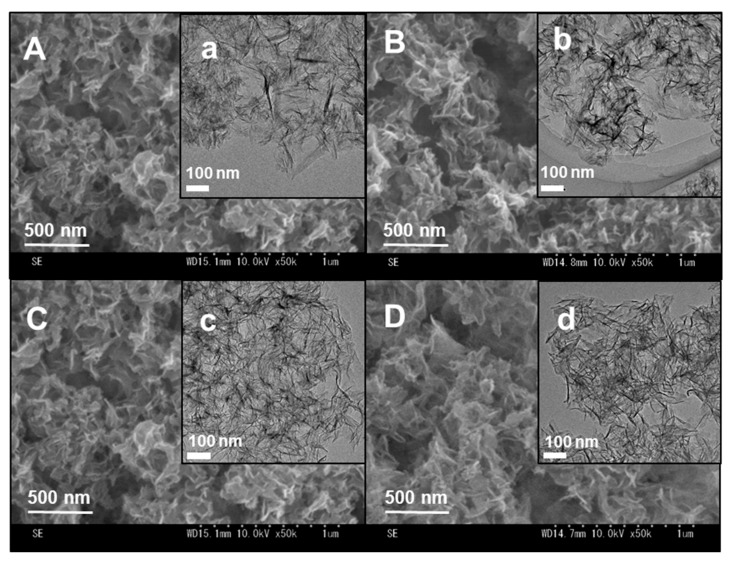
(**A**–**D**) Field-emission scanning electron microscopy (FE-SEM) and (**a**–**d**) transmission electron microscopy (TEM) images of the bovine serum albumin (BSA)–peptide–calcium phosphate (CaP) composites: (**A**, **a**) BSA–CaP, (**B**, **b**) BSA–αpLys–CaP, (**C**, **c**) BSA–εpLys–CaP, and (**D**, **d**) BSA–pArg–CaP.

The morphologies of the bovine serum albumin (BSA)–peptide–CaP composites were analyzed using field-emission scanning electron microscopy (FE-SEM) (Figure 1A–D and transmission electron microscopy (TEM) (Figure 1a–d). Thin sheets of particles were observed, where the particle sizes were approximately 200 × 200 nm in length and 10 nm in thickness. In addition, there were no significant differences in the morphologies of the protein–peptide–CaP composites in comparison with CaP containing no protein. In our previous study [29], αpLys–CaP composites were obtained with rod-like morphologies, formed using a similar synthetic procedure to that described in this work. The protein molecules were found to be affected by the morphologies of the sheet-like CaP materials. 

**Figure 2:**
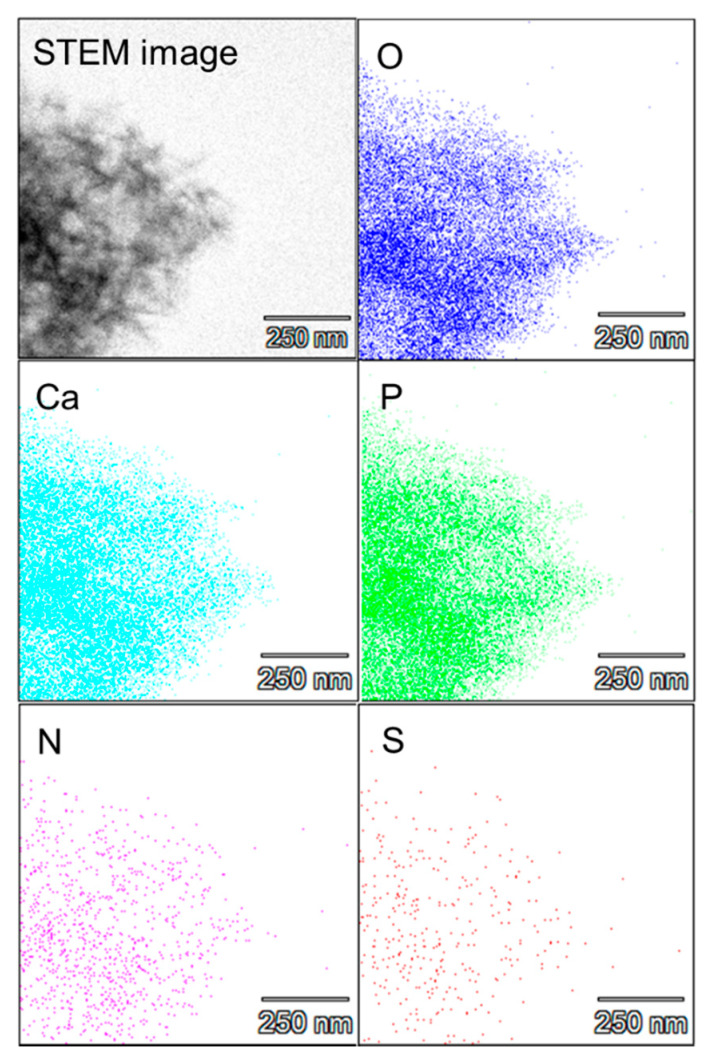
Scanning TEM (STEM)-energy dispersive X-ray spectrometer (EDX) elemental mapping images of BSA–αpLys–CaP. Blue, light blue, green, pink, and orange represent oxygen, calcium, phosphorus, nitrogen, and sulfur, respectively.

Scanning TEM (STEM) elemental mapping experiments were carried out on the BSA–αpLys–CaP composite and the results are shown in Figure 2. The distributions of the five elements, including Ca and P from calcium phosphate, were identical in all STEM images. Nitrogen (N) and sulfur (S) were observed in the composites, where N is found in both peptide and protein, whereas S is found only in the protein. In addition, there was less N and S than the other three elements, in a uniform distribution, confirming that the protein and peptide were homogeneously encapsulated in the CaP. Next, fluorescence microscopy was carried out and the images of the FITC–BSA–αpLys–CaP composite are shown in Figure S2. The imaged material that was 50–100 µm in size was made up of aggregates of nano-sized particles, visualized using the green fluorescent dye FITC. 

**Figure 3:**
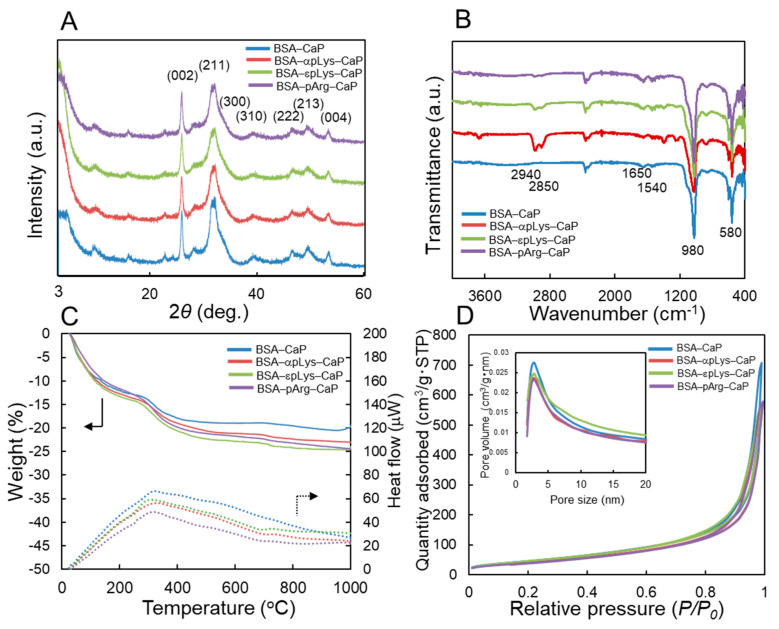
(**A**) Powder X-ray diffractometer (PXRD) patterns, (**B**) Fourier-transform infrared spectra (FTIR) spectra, (**C**) thermogravimetry differential thermal analysis (TG-DTA) curves, and (**D**) nitrogen adsorption–desorption isotherms and pore size distributions (inset) of the BSA–peptide–CaP composites.

The powder X-ray diffractometer (PXRD) patterns of the BSA–peptide–CaP composites are exhibited in Figure 3A. The patterns shown diffraction peaks at 2θ = 26.1°, 31.8°, 32.2°, 39.8°, 46.7°, 49.5°, and 53.6°, which can be attributed to the (002), (211), (300), (310), (222), (213), and (004) planes, respectively, of hydroxyapatite, [Ca_10_(PO_4_)_6_(OH)_2_, HAp, Joint Committee on Powder Diffraction Standards (JCPDS) card number 9–0432] [31]. Judging from the diffraction peaks, all the composites prepared using the present conditions could be identified as HAp, with similar and low crystallinities, indicative that the peptide structures slightly affect the crystallinity of the HAp. It was also considered that HAp preferentially forms on the surface of BSA or BSA–peptide mixtures because there was no strong peak detected at 2θ = 4.7° for octacalcium phosphate [Ca_8_H_2_(PO_4_)_6_·5H_2_O, OCP, JCPDS card number 26–1056] [32]. Iijima et al. described [33] that the addition of 5% BSA strongly reduced the precipitation of OCP. In addition, the Ca/P molar ratios of the BSA–peptide–CaP composites were determined using inductively coupled plasma optical emission spectrometry (ICP-OES), and the results are shown in Table 1. All Ca/P ratios were similar, at 1.37, 1.43, 1.40, and 1.39 for BSA–CaP, BSA–αpLys–CaP, BSA–εpLys–CaP, and BSA–pArg–CaP, respectively, all lower than the stoichiometric ratio of HAp of 1.67. These results also reveal that the BSA–peptide–CaP composites contain low-crystallinity hydroxyapatite (HAp).

Fourier-transform infrared spectra (FTIR) analysis was carried out to confirm the presence of peptide and BSA in the CaP composites (Figure 3B). Characteristic bands of PO_4_^3−^ at 580 and 980 cm^−1^ were observed, assigned to its bending and symmetrical stretching vibrations, respectively. The bands at 1650 and 1540 cm^−1^ can be attributed to the stretching vibration of C=O (amide I), and that at 1540 cm^−1^ to the bending vibration of N–H (amide II), whereas the bands at 2850 and 2940 cm^−1^ correspond to the stretching vibrations of the C–H groups in the protein and peptide, and two peaks of the three peptide-containing CaP composites were more intense than those of the composite with no peptide (BSA–CaP). These results exhibit that the BSA–peptide–CaP composites contain peptide, protein, and CaP. 

Figure 3C indicates the relative amount of BSA–peptide in the composites determined by carrying out thermogravimetry differential thermal analysis (TG-DTA) measurements. The weight losses observed from 200 to 600 °C (judging from DTA curves) correspond to the loss of protein and peptide. The relative amounts of the BSA–peptides were 98% (BSA–CaP), 85% (BSA–αpLys–CaP), 93% (BSA–εpLys–CaP), and 92% (BSA–pArg–CaP), indicating that BSA and the peptides were effectively encapsulated alongside the simultaneous precipitation of CaP. Nitrogen adsorption–desorption isotherms and pore size distribution curves were obtained using the Brunauer–Emmett–Teller (BET) and Barrett–Joyner–Halenda (BJH) methods for the composites, the results of which are shown in Figure 3D and listed in Table 1. All of the composites were found to have high specific surface areas (155–175 m^2^/g) and pore volumes (0.88–1.10 cm^3^/g), with interparticle pores of approximately 4 nm in size, exhibiting that the prepared composites have similar particle characteristics.

The surface charges of all of the BSA–peptide–CaP composites are summarized in Table 1. The surface potential of BSA–CaP was measured as −27.5 mV, because BSA and CaP are negatively charged in pH 7 phosphate buffer. After the incorporation of peptides, the ζ-potentials changed to −11.6 mV (BSA–αpLys–CaP), −4.5 mV (BSA–εpLys–CaP), and −11.2 mV (BSA–pArg) in the case of the composites containing positively charged polypeptide, pLys or pArg, suggesting that the peptide is located close to the surface of the CaP particles.

### 2.2. Controlled Release of Protein from the Protein–Peptide–CaP Composites

**Figure 4:**
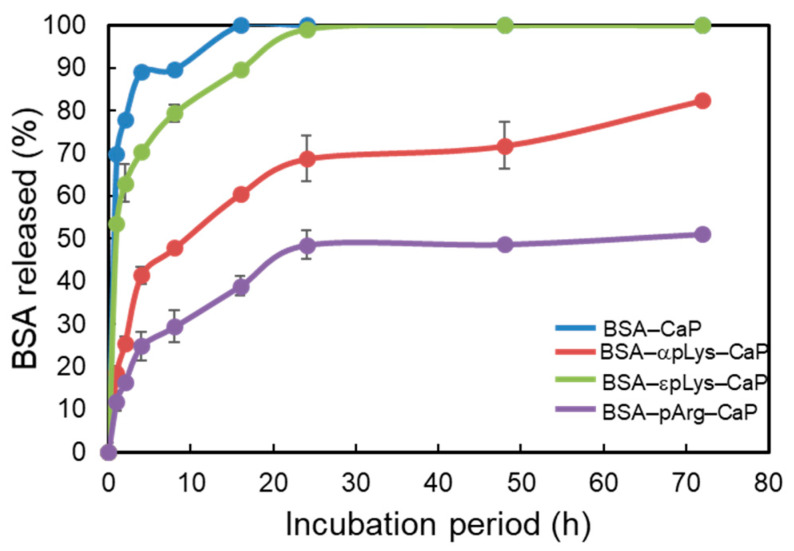
Release profiles, expressed as the percentage of cumulative FITC–BSA released over the course of different incubation periods (2, 4, 6, 8, 16, 24, 48, and 72 h) of the FITC–BSA–peptide–CaP composites.

Figure 4 shows the protein release from the FITC–BSA–CaP composites with different peptides, expressed as the percentage of the cumulative BSA released with respect to the initial amount of FITC–BSA added, as a function of the incubation time (72 h). Comparing the plots in Figure 4, it can be seen that there was a marked burst effect noted in the case of the non-peptide FITC–BSA–CaP and FITC–BSA–εpLys–CaP composites, where over 50% of the total FITC–BSA in the composites was released during the 2 h of incubation. Complete release (100% cumulative release) occurred quickly within 24 h of the test period. As expected, the two peptide-containing composites, FITC–BSA–αpLys–CaP and FITC–BSA–pArg–CaP, showed slower and more sustained release of BSA. In terms of protein delivery from the FITC–BSA–CaP composites containing αpLys and pArg, less of a burst effect was observed. Both composites showed a percentage cumulative release of approximately 25–46% over the 10 h incubation period. The αpLys-containing composite showed a faster increase in the amount of protein in the buffer (42% cumulative release) over the 6 h incubation time. The carrier containing pArg showed better controlled release than the other composites, with only approximately 24% cumulative release being recorded over the same incubation time. From the data on the 16 h of incubation, the peptides showed different profiles, although similar trends were observed up to the end of the incubation (72 h). The amount of FITC–BSA released from the pArg-containing composite into the buffer at different time intervals was lower compared to that released from the αpLys-containing composite. The final cumulative release after 72 h reached 80% and 52% for αpLys and pArg, respectively. In addition, no calcium ions were detected in the solution after soaking all the composites for 72 h, indicative that calcium phosphate did not dissolve under the chosen release conditions (100 mM pH 7 phosphate buffer). Moreover, all BSA encapsulated inside the four composites was released within 3 h in 100 mM pH 5 acetate buffer. From these results, it was determined that the electrostatic interactions between cationic functional groups on the side chain of peptide and anionic groups on the surface of the protein BSA greatly influenced the protein release characteristics of the composites under the studied conditions.

### 2.3. Biocompatibility of the Protein–Peptide–CaP Composites

**Figure 5:**
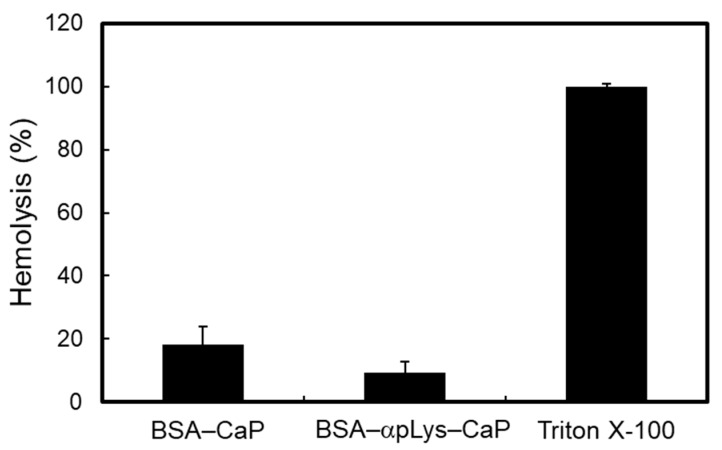
Hemolysis assay in the presence of the composites determined from the release of hemoglobin at concentrations of 2 mg/ml of BSA–CaP and BSA–αpLys–BSA in 1 mL of a 2% RBC suspension at 37 °C over 3 h. Perfect cell lysis was achieved in a 1% Triton X-100 solution.

Two composites (BSA–αpLys–CaP and BSA–CaP) were tested for hemocompatibility using rabbit red blood cells (RBC) (Figure 5), where it was found that Triton X-100 (1%) as a positive control led to 100% RBC lysis. These composites induced low hemolysis activity when they were of sufficiently high concentration (2 mg in 1 mL of a 2% RBC suspension), increasing the amount of free hemoglobin from the RBCs present in the supernatant to 18% and 8% at the above concentration, suggesting that the BSA–peptide–CaP composites did not influence the RBC and good hemocompatibility.

**Figure 6:**
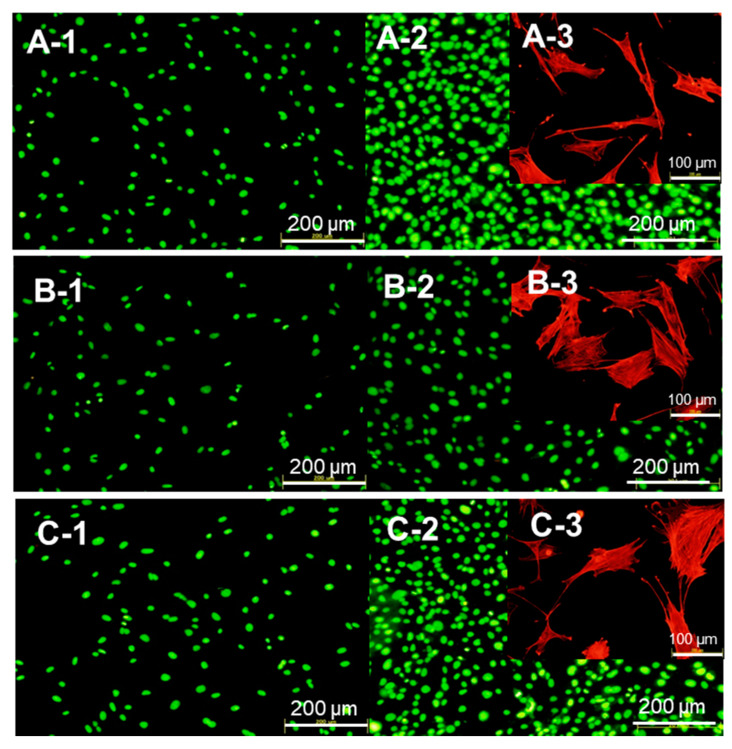
Evaluation of cell viability after culturing for 24 h (**A-1**–**C-1**) and 72 h (**A-2**–**C-2**) with the composites (A: medium without the composites, B: BSA–CaP, C: BSA–αpLys–CaP) by staining with Cyto dye, a cell-permeable green fluorescent dye for staining live cells, and propidium iodine (PI), a non-cell permeable red fluorescent dye for staining dead cells. Fluorescence microscopy images of the actin filaments of cells co-cultured with the composites (**A-3**–**C-3**). Actin fibers are stained in red.

**Figure 7:**
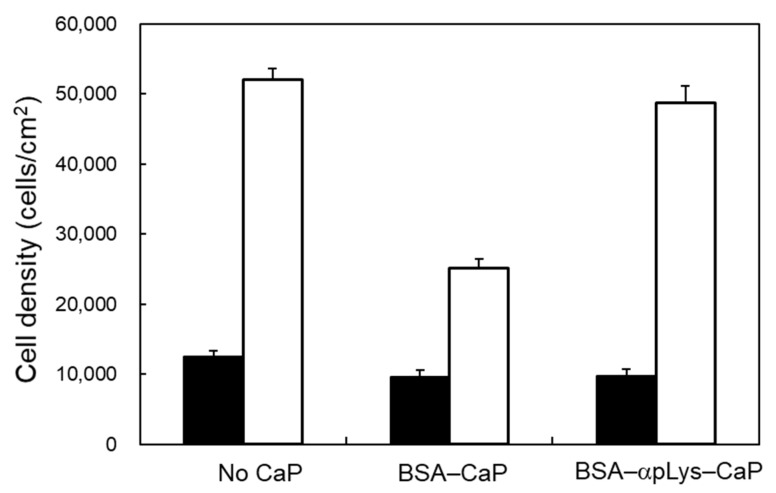
Cell proliferation after culturing for 24 h (black bars) and 72 h (white bars) with the protein–peptide–CaP composites. Cell numbers (density), which are represented as values per unit area, were evaluated using the ImageJ software.

Figure 6 A-1–C-1 show mouse osteoblast-like MC3T3-E1 cell viability and proliferation after 24 h of their co-culturing with the BSA–CaP and BSA–αpLys–CaP composites. The cell densities of both composites were similar after 24 h, suggesting that the protein–peptide–CaP composites did not negatively affect the initial attachment of these cells. The viability and proliferation of these cells were explored using a LIVE/DEAD double staining method, and their cell viability was determined using green staining for living cells and red for dead cells over culturing periods of 24 h, where after this period, no dead cells were observed. Consequently, the cell viability of the long-term co-cultures (72 h) with the composites was perfect (Figure 6A-2–C-2). The cell proliferation rate observed with the BSA–αpLys–CaP composite had a similar cell density (approximately 50,000 cells/cm^2^) to that of the medium with no added composite used as a positive control (Figure 6). In contrast, as can be seen in Figure 7, the density of cells with the BSA–CaP composite showed approximately 50% decrease in growth compared to those with BSA–αpLys–CaP. Figure 6A-3–C-3 show fluorescence micrographs of actin stress fibers in cells co-cultured with BSA–CaP and BSA–αpLys–CaP. Compared to the medium without any composite used as a positive control, in the other samples, the cultured cells spread in a planar shape and the formation of actin stress fibers with many cell pseudopods was clearly observed inside cells with the BSA–αpLys–CaP composite. As can be seen in Figure 6C-3, actin stress fibers were visible inside elongated shaped cells, however, fewer cell pseudopods formed co-cultures with the non-peptide BSA–CaP composite. These results suggest that cell spreading and proliferation can strongly improve in response to peptide-containing CaP composites. It has been reported that cationic polypeptides such as pArg- and pLys-based materials have high cell penetrating ability and therefore, their inorganic or organic composites are expected to make high performance drug delivery materials [34,35,36]. 

## 3. Materials and Methods 

### 3.1. Materials

Calcium acetate monohydrate [(CH_3_COO)_2_Ca·H_2_O], diammonium hydrogen phosphate [(NH_4_)_2_HPO_4_], and phosphate buffered saline (PBS)(−) were obtained from FUJIFILM Wako Pure Chemical Co. (Osaka, Japan). αPLys [molecular weight (Mw) = 15,000–30,000 g/mol], αpArg (MW = 15,000–70,000 g/mol). FITC–BSA, BSA, RBC, and a LIVE/DEAD double staining kit were purchased from Merck KGaA (Darmstadt, Germany). ε-PLys with a Mw of 4000 g/mol was gifted by JNC Co., (Tokyo, Japan). A calcium colorimetric assay kit was obtained from Metallogenics Co. (Chiba, Japan). Minimum essential alpha medium (α-MEM), fetal bovine serum (FBS), penicillin–streptomycin solution (×100), trypsin/ethylenediaminetetraacetic acid (EDTA) solution, and rhodamine–phalloidin were obtained from Thermo Fisher Scientific Inc. (Waltham, MA, USA). All reagents were of analytical grade and were used without further purification.

### 3.2. Preparation of the Protein–Peptide–Calcium Phosphate (CaP) Composites

The protein–peptide–calcium phosphate composites were prepared as follows. First, 10 mg of polypeptide was dissolved in 200 mL of a 15 mM (CH_3_COO)_2_Ca solution, and the mixture was stirred for 15 min at 27 °C. Then, 10 mg of FITC–BSA for the release tests or BSA for the cell culture tests was added to solution, which was then stirred for a further 30 min at 27 °C. Next, 200 mL of a 9 mM (NH_4_)_2_HPO_4_ solution was added to the mixture, and the mixture was stirred for a further 3 h at the same temperature. The obtained solids products were collected via centrifugation at 6000 rpm for 10 min, washed with deionized water, and then freeze-dried before further use. For example, the composite prepared from BSA, αpLys, and calcium phosphate was named as BSA–αpLys–CaP.

### 3.3. Characterization of the Protein–Peptide–Calcium Phosphate (CaP) Composites

The morphologies of the protein–peptide–CaP composites were examined using FE-SEM (Hitachi S-4700, Hitachi, Tokyo, Japan), operating at an accelerating voltage of 10 kV, and TEM (JEM-2010, JEOL Co., Tokyo, Japan) operating at 200 kV. The elemental distributions of phosphorous, calcium, and oxygen (from the calcium phosphate), and nitrogen and sulfur (from the protein and peptide) were measured using a STEM operating (JEM-2010, JEOL Co., Tokyo, Japan) at an acceleration voltage of 200 kV, equipped with an energy dispersive X-ray spectrometer (EDX; Pathfinder version 1.4.11, Thermo Fisher Scientific Inc. Waltham, MA, USA). The crystal structures data of the samples were recorded using a PXRD (Smart Lab SE/B1, Rigaku Co., Tokyo, Japan), equipped with a CuKα radiation source with a wavelength of 0.1541 nm in the range of 3°–60°. FTIR were recorded using a JASCO spectrometer (MFT-2000, Tokyo, Japan) in the range of 400–4000 cm^−1^ using KBr disks (sample/KBr = 1:100). TG-DTA experiments were carried out using Thermo Plus TG 8120 apparatus (Rigaku Co., Tokyo, Japan) in a Pt crucible at heating rates of 10 °C/min, from room temperature to 1000 °C. The weight losses from 250 to 600 °C were assigned to the loss of protein and peptide from the composite. The pore sizes and distributions of the composites were characterized from BET analysis. The nitrogen adsorption–desorption isotherm curves of the composites were recorded using a specific surface and pore analyzer (Micromeritics TriStar 3000, Shimadzu Co., Tokyo, Japan). Using the BJH model, the pore volumes and pore size (>1.7 nm) distributions were obtained from the desorption curves of the isotherms. The zeta (ζ) potentials of the composites at a concentration of 2.0 mg/mL in 100 mM phosphate buffer (pH 7.0) were measured using an ELSZ-2 zeta potential analyzer (Otsuka Electronics Co., Tokyo, Japan). The molar ratios of calcium to phosphate (Ca/P ratio) in the composites were determined via ICP-OES (IRIS Advantage, Thermo Fisher Scientific Inc., Waltham, MA, USA).

### 3.4. Protein Release from the Protein–Peptide–CaP Composites

The amount of protein (FITC–BSA) released from the composites was determined by measuring the fluorescence intensity of the supernatants after incubation of the composite in 100 mM phosphate buffer (pH 7) for 2, 4, 6, 8, 16, 24, 48, or 72 h. An amount of 2 mg of each of the protein–peptide–calcium phosphate composites was added to 1 mL of 100 mM pH 7.0 phosphate buffer at 37 °C for each incubation period. After completion of the incubation period, the mixtures were centrifuged at 12,000 rpm for 5 min, and the supernatants were measured using spectrofluorophotometer (RF-5300PC, Shimadzu Co., Kyoto, Japan). The fluorescence emission spectra of the FITC-bound BSA from each sample were recorded at excitation and fluorescence wavelengths of 498 and 522 nm, respectively.

### 3.5. Hemocompatibility Assay

The hemocompatibility assay of the composites was performed by measuring the hemoglobin release from rabbit RBC [37]. A 5% suspension of RBC in 100 mM pH 7.0 phosphate buffer was used for each assay. An amount of 2 mg of the dispersions of the composites was incubated in 1 mL of a 2% RBC suspension at 37 °C for 3 h. After incubation, the suspensions were centrifuged at 12,000 rpm for 5 min, and the supernatants were used in the measurements. The amount of free hemoglobin contained in the supernatant was measured via ultraviolet-visible (UV-Vis) absorption spectroscopy (V730, JASCO, Co., Tokyo, Japan) at 595 nm. Triton X-100 (1%) was used for the hemoglobin release of 100% lysis.

### 3.6. Cell Behavior After Being Cultured with the Protein–Peptide–CaP Composites

Mouse osteoblast-like MC3T3-E1 cells (Riken Bio Research Center, Tsukuba, Japan) were cultured in α-MEM supplemented with 10 wt% FBS and 1 wt% penicillin–streptomycin [38]. The cell cultures were maintained in an incubator equilibrated with 5% carbon dioxide (CO_2_) at 37 °C. Confluent MC3T3-E1 cells were trypsinized and a suspension of 0.30 × 10^5^ cells/mL was added to each well. After an incubation period of 6 h, the culture medium was replaced with test medium containing 0.1 mg/mL of each of the BSA–peptide–CaP composites, and was then incubated for a further 24 and 72 h to perform two types of cell staining techniques, LIVE/DEAD double staining and actin filament staining using rhodamine–phalloidin. A LIVE/DEAD double staining kit was used to quantify the viability of cells cultured with the composites. The cells were stained using Cyto dye, a cell-permeable green fluorescent dye that is used for live cells, and propidium iodide (PI), a non-cell-permeable red fluorescent dye that is used for dead cells. The cultured cells were fixed using 3.7% formaldehyde in PBS(−), and then permeabilized with 0.1% Triton-X100 in PBS(−) to achieve the rhodamine–phalloidin labeling of actin filaments. The stained samples were immediately observed using a fluorescence microscope BZ-X800, KEYENCE, Osaka, Japan) equipped with a band-pass filter.

## 4. Conclusions

BSA–peptide–CaP composites were prepared using the relatively simple procedure of encapsulation during the co-precipitation of calcium phosphate with protein–peptide hybrids. After characterization of the composites using a variety of techniques, it was found that the protein–peptide hybrids could be effectively loaded inside the CaP structure. The in vitro release assays revealed that the composite without peptide let to burst protein release, however, upon the addition of peptide, the release profiles gradually changed during the release tests. The structures of the peptides greatly affect the release profile, and the composites with polyarginine were the most efficient in terms of promoting the sustainable release of protein from the composites. The release analysis suggested that peptide–protein interactions were mainly responsible for the formation of the protein delivery system. In cell tests using mouse osteoblast-like MC3T3-E1 cells, the cell spread and proliferation when co-cultured with protein–peptide–CaP showed a similar trend to a medium without any composite, however, when the protein–CaP composite containing no peptide was used, restricted cell spread and proliferation was observed. In addition, the protein–peptide–CaP composites also displayed high hemocompatibility. These encouraging findings open up novel and interesting prospects for the preparation methods of protein–peptide–CaP composites and the synthesis of new potential materials with high biocompatibility for use as drug release systems. Additional studies on the ability of protein–peptide–CaP composites to deliver protein to cells are currently in progress.

## Figures and Tables

**Table 1:** ζ-Potentials, Ca/P molar ratios, and nitrogen physisorption parameters of the BSA–peptide–CaP composites.

BSA–Peptide–CaP Composites	ζ-Potential ^a^ (mV)	Ca/P Molar Ratio ^b^	Pore Volume ^c^ (cm^3^/g)	Specific Surface Area ^c^ (m^2^/g)
BSA–CaP	−27.5	1.37	1.10	174.9
BSA–αpLys–CaP	−11.6	1.34	0.88	155.3
BSA–εpLys–CaP	−4.5	1.33	0.88	178.3
BSA–pArg–CaP	−11.2	1.32	0.90	147.6

^a^ The ζ-potentials of the BSA–peptide–CaP composites were obtained via an electrophoretic light scattering method. The particles were dispersed in 100 mM phosphate buffer (pH 7.0) with sonication for 3 min before measurements were carried out. ^b^ The Ca/P molar ratios were measured via inductively coupled plasma optical emission spectrometry (ICP-OES). ^c^ The specific surface areas and pore volumes of the BSA–peptide–CaP composites were calculated on the basis of the nitrogen adsorption–desorption isotherms using the Brunauer–Emmett–Teller (BET) and Barrett–Joyner–Halenda (BJH) methods.

## Supplementary Materials

The following are available online, Figure S1: UV-Vis spectra of the bovine serum albumin (BSA) solution before encapsulation in the CaP material (solid line) and the supernatant after the precipitation of the BSA–CaP composite (dotted line), Figure S2: Fluorescence microscopy image of the FITC–BSA–αpLys–CaP composite. Nano-sized composites were aggregated and visualized by fluorescent dye-conjugated BSA.
