# Supplementary material for: Preparation of Protein–Peptide–Calcium Phosphate Composites for Controlled Protein Release
Source: Molecules. 2020 May 14;25(10):2312. doi: 10.3390/molecules25102312 (PMC7287863; doi:10.3390/molecules25102312)
Supplement: Supplementary file 1 [file molecules-25-02312-s001.pdf]

Supplementary materials

Preparation of protein–peptide–calcium composites for controlled protein release

Katsuya Kato*, Sungho Lee, Fukue Nagata

National Institute of Advanced Industrial Science and Technology (AIST), 2266-98 Anagahora, Shimoshidami, Moriyama-ku, Nagoya 463-8560, Japan

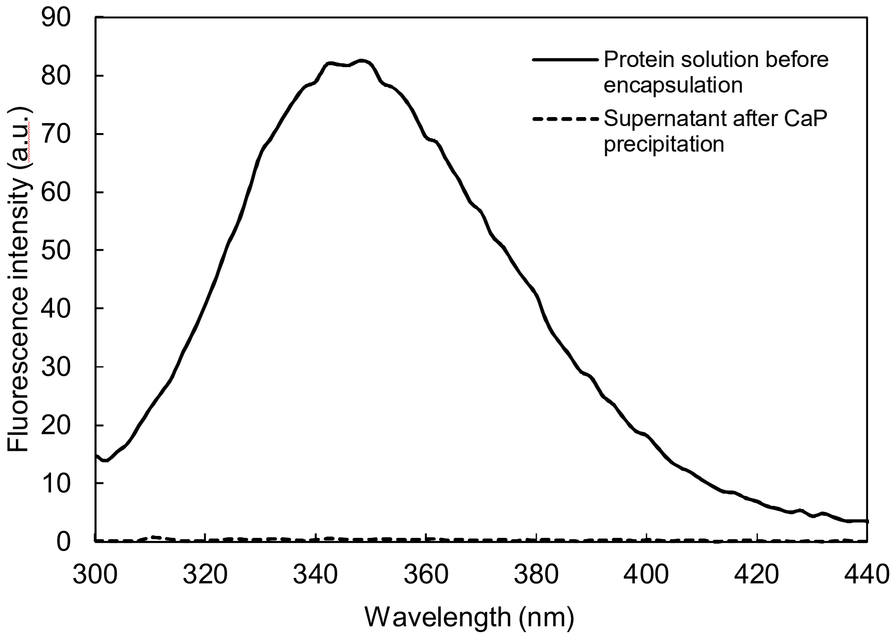

Figure S1. UV-vis spectra of the bovine serum albumin (BSA) solution before encapsulation in the CaP material (solid line) and the supernatant after the precipitation of the BSA–CaP composite (dotted line).

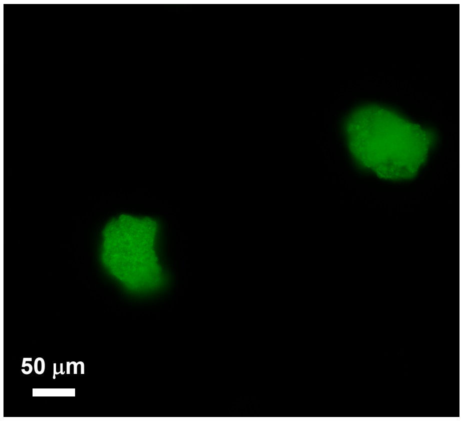

Figure S2. Fluorescence microscopy image of the FITC–BSA–αpLys–CaP composite. Nano-sized composites were aggregated and visualized by fluorescent dye-conjugated BSA.
